# Reconstructing rodent brain signals during euthanasia with eigensystem realization algorithm (ERA)

**DOI:** 10.1038/s41598-024-61706-y

**Published:** 2024-05-28

**Authors:** Khitam Aqel, Zhen Wang, Yuan B. Peng, Pedro D. Maia

**Affiliations:** 1 Department of Mathematics, University of Texas at Arlington, 411 S Nedderman Dr, Arlington, TX 76019 USA; 2 Department of Psychology, University of Texas at Arlington, 501 S Nedderman Dr, Arlington, TX 76019 USA

**Keywords:** Bayesian estimator of abrupt change, Seasonality, and Trend (BEAST), Brain, Eigensystem realization algorithm (ERA), Euthanasia, Local field potential (LFP), Model reduction, Psychology, Diseases, Applied mathematics, Computational science, Scientific data, Statistics, Mathematics and computing, Software, Neuroscience, Computational neuroscience

## Abstract

We accurately reconstruct the Local Field Potential time series obtained from anesthetized and awake rats, both before and during CO_2_ euthanasia. We apply the Eigensystem Realization Algorithm to identify an underlying linear dynamical system capable of generating the observed data. Time series exhibiting more intricate dynamics typically lead to systems of higher dimensions, offering a means to assess the complexity of the brain throughout various phases of the experiment. Our results indicate that anesthetized brains possess complexity levels similar to awake brains before CO_2_ administration. This resemblance undergoes significant changes following euthanization, as signals from the awake brain display a more resilient complexity profile, implying a state of heightened neuronal activity or a last fight response during the euthanasia process. In contrast, anesthetized brains seem to enter a more subdued state early on. Our data-driven techniques can likely be applied to a broader range of electrophysiological recording modalities.

## Introduction

Understanding complex biological and physical processes has long relied on nonlinear systems and control theory^1^. In recent years, data-driven approaches have emerged as powerful alternatives to traditional methods, offering mathematically simpler equations to describe observed data^2,3^. These approaches enable the reconstruction of intricate signals using large linear systems. In this study, we apply such data-driven techniques to analyze Local Field Potential (LFP) signals recorded from the brains of both anesthetized and freely moving rats, both before and during CO_2_ euthanasia. Our aim is to gain insights into the neural mechanisms underlying the transition to death.

Data-driven techniques typically derive low-dimensional input-output models that reveal the modal characteristics of a system using time-domain data, all without prior knowledge of the system’s underlying model^4–12^. Our approach assumes that the limited available LFP signals are driven by a larger, unobserved full system, such as the entire brain. To find this system, we employ the Eigensystem Realization Algorithm (ERA), a method that constructs low-rank approximations of the full system by applying Singular Value Decomposition (SVD) to the Hankel matrix created from the time-series data^4,5^. By projecting the coherent structure of the higher-dimensional dynamical system onto a smaller set of modes, we can efficiently reconstruct LFP signals recorded from various brain regions.

The LFP data utilized in this study originates from a previous rodent study on migraine^13,14^. These LFP recordings were obtained from four specific brain regions known to be involved in the pathophysiology of migraine headaches^14–16^. Recording LFPs from multiple brain regions is technically challenging, especially given the high sampling rate and extended recording durations, resulting in lengthy time series data. To handle the sheer volume of data points, we employ the Bayesian Estimator of Abrupt Change, Seasonality, and Trend (BEAST)^17–19^, a statistical method designed for segmented regression^17^. The BEAST method effectively segments the time series by identifying crucial change points, simplifying the representation of the data for subsequent reconstruction using ERA.

The synergy of these techniques yields high-quality LFP reconstructions and provides an estimate of the system’s complexity, as measured by the dimensionality of the presumed higher-dimensional space from which the time series originates. This paper presents a novel application of data-driven methodologies to LFP signals, shedding light on the complex neural dynamics underlying euthanasia-induced transitions in brain activity. Furthermore, our methodology is likely to be applicable to other types of electrophysiological recording modalities and neural settings.

**Figure 1:**
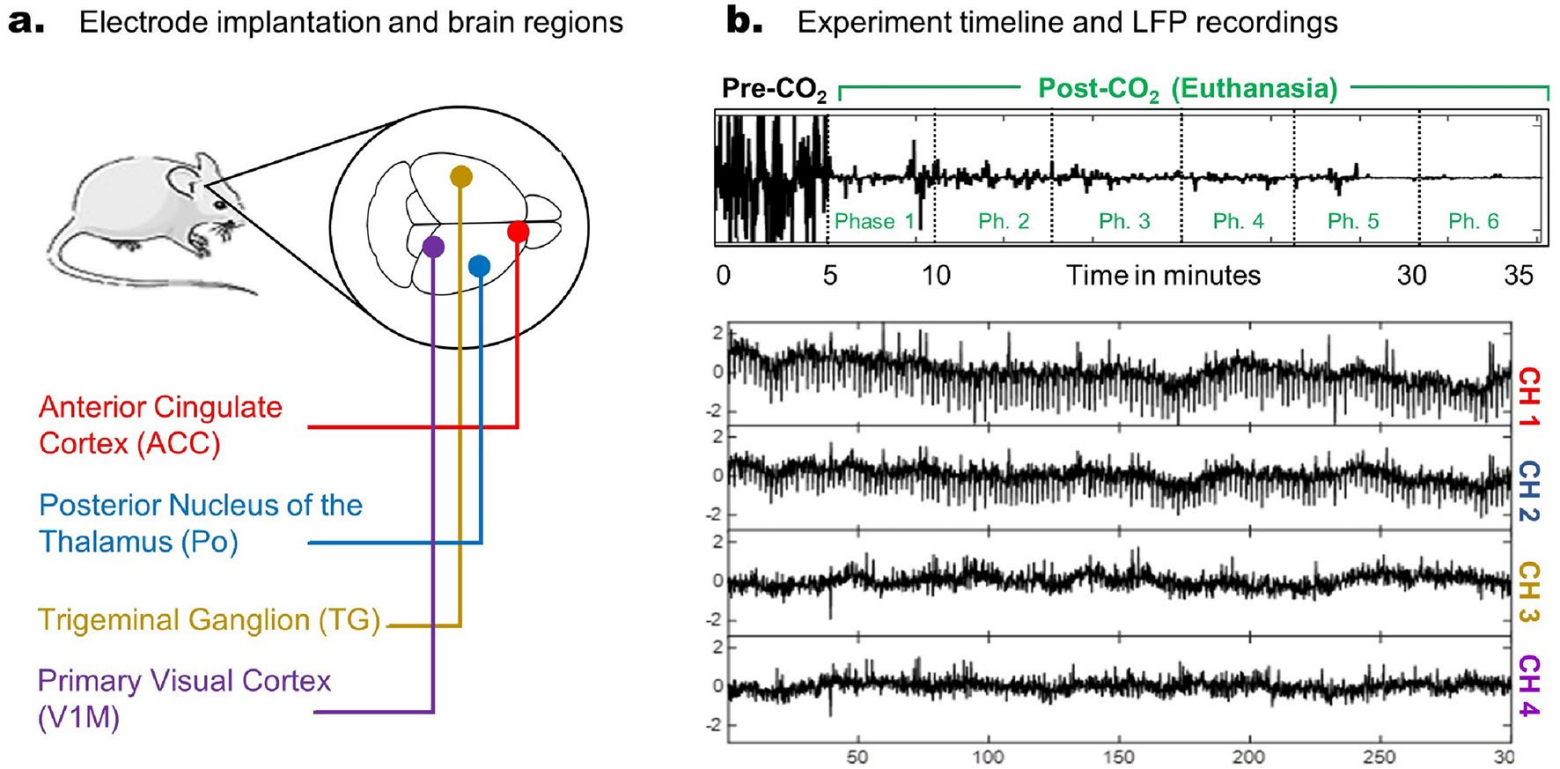
An overview of the experimental setup of the LFP recordings from rodent brains before and during euthanasia. Panel (**a**) shows the approximate locations of four LFP electrodes implanted in the rats^14^: the right anterior cingulate cortex (Ch.1, in red), the right posterior nucleus of the thalamus (Ch. 2, in blue), the left trigeminal ganglion (Ch.3, in yellow), and the right primary visual cortex (Ch.4, in purple). Panel (**b**) illustrates the experimental timeline and an example of the LFP recordings. The first 5 min (Pre-CO_2_) typically display stronger activity, which decays throughout the continuous delivery of CO_2_ administered at 100% concentration (euthanasia). In our analyses, the LFP recordings were divided into Phases of 5-min duration each.

## Materials and methods

### Animals and experimental procedures

#### Moving-awake (AW) versus anesthetized (AN) rats

The experimental data for this study originated from a previous rodent study on migraines^14^. We utilized ten male Sprague Dawley rats, each weighing between 300 an d 400 grams, and divided them into two groups: freely moving-awake (AW) and anesthetized (AN), with five rats in each group. For anesthesia, we administered isoflurane at a 5% concentration for induction, maintaining it between 1.5 an d 3% throughout the experiments. The rats were housed in an Animal Care Facility, subject to a 12-hour light/dark cycle, with continuous access to water and food pellets. The Institutional Animal Care and Use Committee (IACUC) at the University of Texas at Arlington approved all experimental procedures, and all methods were carried out in strict accordance with institutional guidelines and regulations.

#### Location of implanted LFP electrodes

Based on the Paxinos and Watson brain atlas (sixth edition)^16^, four bipolar stainless-steel electrodes (0.254 mm diameter) were implanted at specific brain regions: the right anterior cingulate cortex (ACC), right posterior nucleus of the thalamus (Po), left trigeminal ganglion (TG), and right primary visual cortex (V1M), as shown in Fig. 1a. The coordinates for electrode placement were ACC (0 mm posterior, 0.70 mm lateral right, 3.20 mm deep), Po (3.72 mm posterior, 2.20 mm lateral right, 5.60 mm deep), TG (4.30 mm posterior, 3.40 mm lateral left, 10.00 mm deep), and V1M (7.44 mm posterior, 3.40 mm lateral right, 1.60 mm deep). A ground and reference screw was placed under the skull, and dental cement along with anchor screws were used to secure the electrodes. See^14^ for details.

#### CO_2_ administration and clinical death

As depicted in Fig. 1b, euthanasia commenced following 5 min of baseline LFP recordings, during which 100% concentration CO_2_ was continuously administered. Clinical death was confirmed between 5 and 7 min after the onset of CO_2_ administration, characterized by the absence of spontaneous respiration and the paws turning pale and white, indicative of ceased blood flow.

### Data pre-processing

#### Partitioning long recordings into phases

As illustrated in Fig. 1b, we divided the 35-min-long recording into 5-min phases, each containing approximately 900 k time points. The first phase was recorded before CO2 to establish a baseline for both groups, while the subsequent phases (phases 1–6) were recorded during euthanasia (5–35 min). This division became necessary to manage the large size of the original dataset resulting from a sampling rate of 3000 Hz. For example, the entire LFP recording from anesthetized rat AN1 comprised approximately 6 million time points per channel.

#### Signal denoising

We apply Matlab’s smooth function to each time series. This function is widely used in signal processing and data analysis to reduce noise and enhance data visualization. It applies a moving average filter over a predefined window size, with each output point being the average of the points within the window.

#### Segmented regression of LFP time series via BEAST

**Figure 2:**
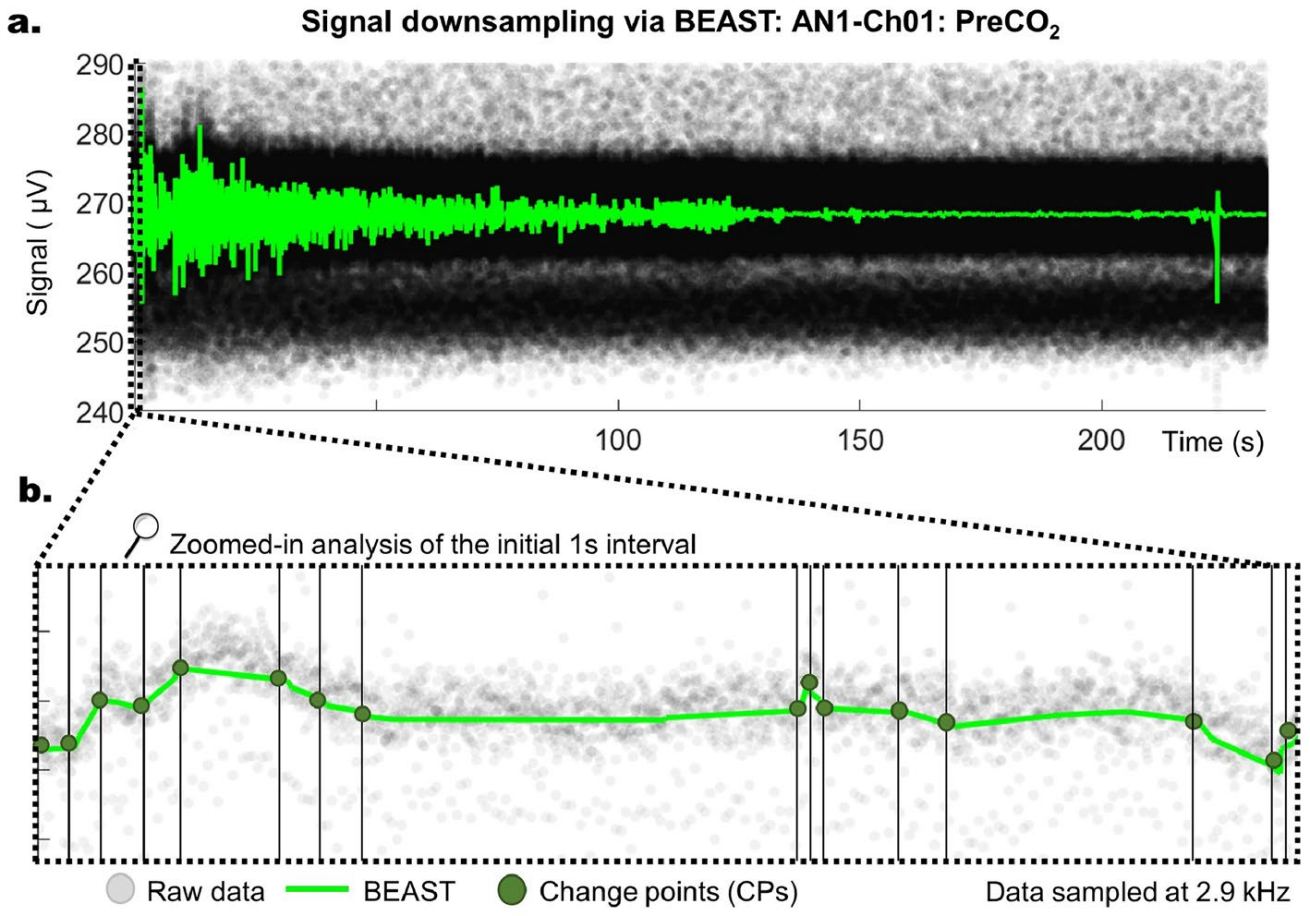
Stereotypical example of LFP time series reconstruction using BEAST. Panel (**a**) displays the original data as black dots and the BEAST reconstruction in green. Panel (**b**) focuses on a shorter time window (initial 1s interval sampled at 2.9 kHz), highlighting detected change points (CPs) as dark green dots, connected by linear segments to illustrate significant trends. Remarkably, capturing the (non-uniform) locations and values of just 16 CPs effectively represents the underlying pattern of thousands of raw data points. Additionally, computing linear segments connecting these points can be straightforwardly performed in subsequent steps if resampling of the signal is required.

The sheer number of data points within each 5-min interval (illustrated in light gray in Fig. 2a) still made time series reconstruction via ERA prohibitive, emphasizing the need for an effective downsampling method. To address this challenge, we utilized the Bayesian Estimator of Abrupt Change, Seasonality, and Trend (BEAST) algorithm to preprocess the extensive LFP data obtained from each rat^17–19^. BEAST is a robust time-series decomposition algorithm that can disentangle time series or sequential data into distinct components, including abrupt changes, trends, and seasonal variations.

BEAST can identify the (non-uniform) locations and values of Change Points (CPs) in the LFP signals. CPs serve as the endpoints of linear segments that approximate the most significant trends in the time series. Figure 2b zooms in on a small data segment to visually demonstrate time series approximation using BEAST, highlighting CPs in dark green. Despite the raw data’s noisy and outlier-laden appearance, discernible trends characterized by denser and darker clusters emerge, which can be effectively approximated using linear segments. By precisely locating CPs at these segments’ endpoints, we can significantly reduce the complexity of the signal representation. In this instance, merely documenting the (non-uniform) location and value of 16 CPs was sufficient to capture the underlying pattern represented by thousands of raw data points. We observe that down-sampling via BEAST is not a mandatory step for using ERA; depending on the dataset, uniform downsampling and/or spline smoothing may suffice. However, in our context, BEAST provided significant advantages over simpler methods. For more information, refer to SI-Fig. 1 and the accompanying text SI-1.

#### Further pre-processing steps

The signals downsampled by BEAST retain only the locations and values of Change Points (CPs), which are frequently misaligned across channels. Nonetheless, linking these CPs with linear segments is straightforward and we interpolate the signal to achieve a coarse uniform discretization that is consistent across all channels. For additional information, see SI-Fig. 2 and the related text in SI-2.

### Eigensystem realization algorithm (ERA)

#### Theoretical background

**Figure 3:**
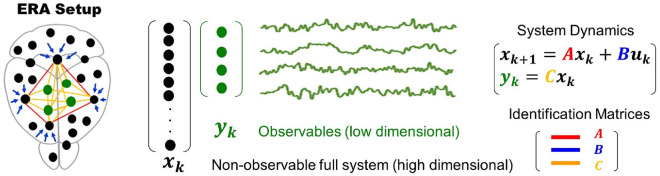
An overview of the ERA setup: ERA assumes that there is an underlying high dimensional, *non-observable* system (e.g. the entire brain) responsible for the overall dynamics. The variables associated with the full system at discrete time *k* are given by the vector $\textbf{x}_{k}$ and represented in black. The *observed* variables are represented in green and given by a low dimensional vector $\textbf{y}_{k}$, with $\text {dim}(\textbf{y}) \ll \text {dim} (\textbf{x})$. To find the governing equations, one must identify the matrices A, B, and C. Note that matrix C models the influence of the full system $\textbf{x}$ onto the observed variables **y.**

We now review a system identification technique known as the Eigensystem Realization Algorithm (ERA) and how we plan to use it to reconstruct the LFP signals. As described in^20^, ERA operates under the assumption that the observed signals can be effectively represented by the following discrete-time dynamical system:

1 $$\begin{aligned}{}&\textbf{x}_{k+1} = A \textbf{x}_{k}+B \textbf{u}_{k}\\&\textbf{y}_{k} = C \textbf{x}_{k} \end{aligned} $$

where $\textbf{x}\in \mathbb {R}^{\text {n}}$ corresponds to the full, non-observable high-dimensional state of the system (e.g., the entire brain), $\textbf{u}\in \mathbb {R}^{\text {n}}$ corresponds to an actuation input, and $\textbf{y}\in \mathbb {R}^{\text {m}}$ correspond to the low dimensional, observable measurements (e.g., the few recorded LFP channels). See Fig. 3 for a schematics. If we assume that

$$\begin{aligned} \textbf{u}_{k}= {\left\{ \begin{array}{ll} 0,&{} \text {for} ~~ k\in \mathbb {Z^+} \\ I,&{} \text {for} ~~ k=0, \end{array}\right. } \end{aligned}$$

and $\textbf{x}_{0} = \textbf{0}$, then the (observable) $\textbf{y}$ measurements are given by the closed-form expression

2 $$\begin{aligned} \textbf{y}_{k}= {\left\{ \begin{array}{ll} CA^{k-1}B,&{} \text {for} ~~ k\in \mathbb {Z^+} \\ 0,&{} \text {for} ~~ k=0. \end{array}\right. } \end{aligned}$$

As a consequence, discovering the system’s governing equations becomes equivalent to finding the matrices *A*, *B*, and *C* solely from the observable time series given by $\textbf{y}_{k}$. In our study, we are unable to access the internal states *x* of the entire brain, and instead, we can only measure the 4-dimensional vector *y* corresponding to the 4-electrodes implanted in the rat’s brain (refer to Fig. 1).

To find the desired matrices from the previous step, we organize the measurements into two Hankel matrices *H* and $H'$, the rows of which are time-shifted impulse-response measurements^20^:

3a $$\begin{aligned} H := \begin{bmatrix} \textbf{y}_1 &{} \textbf{y}_2 &{}\cdots &{} \textbf{y}_{m-s} \\ \textbf{y}_2 &{} \textbf{y}_3 &{}\cdots &{} \textbf{y}_{m-s+1} \\ \vdots &{} \vdots &{}\ddots &{} \vdots \\ \textbf{y}_s &{} \textbf{y}_{s+1} &{}\cdots &{} \textbf{y}_{m-1} \end{bmatrix} = \begin{bmatrix} CB &{} CAB &{}\cdots &{} CA^{m-s-1}B \\ CAB &{} CA^{2}B &{}\cdots &{} CA^{m-s}B \\ \vdots &{} \vdots &{}\ddots &{} \vdots \\ CA^{s-1}B &{} CA^{s}B &{}\cdots &{} CA^{m-2}B \end{bmatrix}, \end{aligned}$$

3b $$\begin{aligned} H' := \begin{bmatrix} \textbf{y}_2 &{} \textbf{y}_3 &{}\cdots &{} \textbf{y}_{m-s+1} \\ \textbf{y}_3 &{} \textbf{y}_4 &{}\cdots &{} \textbf{y}_{m-s+2} \\ \vdots &{} \vdots &{}\ddots &{} \vdots \\ \textbf{y}_{s+1} &{} \textbf{y}_{s+2} &{}\cdots &{} \textbf{y}_{m} \end{bmatrix} = \begin{bmatrix} CAB &{} CA^{2}B &{}\cdots &{} CA^{m-s}B \\ CA^{2}B &{} CA^{3}B &{}\cdots &{} CA^{m-s+1}B \\ \vdots &{} \vdots &{}\ddots &{} \vdots \\ CA^{s}B &{} CA^{s+1}B &{}\cdots &{} CA^{m-1}B \end{bmatrix}, \end{aligned}$$

The singular value decomposition (SVD) of *H* yields

4 $$\begin{aligned} H =U \Sigma V^* \approx U_r \Sigma _r V_r^* \end{aligned}$$

where the subscript *r* denotes a rank-*r* truncation. In a sense, the columns of *U* and *V* are eigen-time-series that best describe the impulse response across various time-scales. Thinking of the eigen-time-series of the previous step as modes, we can use their coefficients as states in a reduced order model, thus obtaining the following low dimensional approximations for *A*, *B* and *C* (see^2,20^):

5 $$ A_{r}  = \Sigma _{r}^{{ - 1/2}} U_{r}^{*} H^{\prime}V_{r} \Sigma _{r}^{{ - 1/2}} ,\,\,B_{r}  = \Sigma _{r}^{{1/2}} V^{*} \left[ {\begin{array}{*{20}c}    {I_{p} } & 0  \\    0 & 0  \\   \end{array} } \right]~,\,\,C_{r}  = \left[ {\begin{array}{*{20}c}    {I_{q} } & 0  \\    0 & 0  \\   \end{array} } \right]U\Sigma _{r}^{{1/2}} , $$

where the identity matrices $I_{p}$ and $I_{q}$ extract the first *p* columns and *q* rows respectively.

#### Number of stacks (NS) and system complexity

The Hankel matrices in Eqs. (3a) and (3b) consist of columns representing time-delayed or time-shifted coordinates, effectively stacking the measurement vector $\textbf{y}$ at the current time alongside copies of $\textbf{y}$ at future times. These Hankel matrices can be viewed as shift-stacked data matrices, encompassing *s* time-shifted state vectors^20^. The determination of the optimal Number of Stacks (NS) for achieving accurate reconstructions hinges on the system’s time-varying behavior. Using more stacks generally reduces error by capturing additional information about the system dynamics. However, practical considerations, such as computational resources, may also influence the choice of NS. In our analysis of brain activity in rats, NS varied according to the experimental phase and the rats’ state (awake or anesthetized). As expected, the number of stacks generally decreased during euthanasia for both groups, a phenomenon we will explore in detail in the following section.

**Figure 4:**
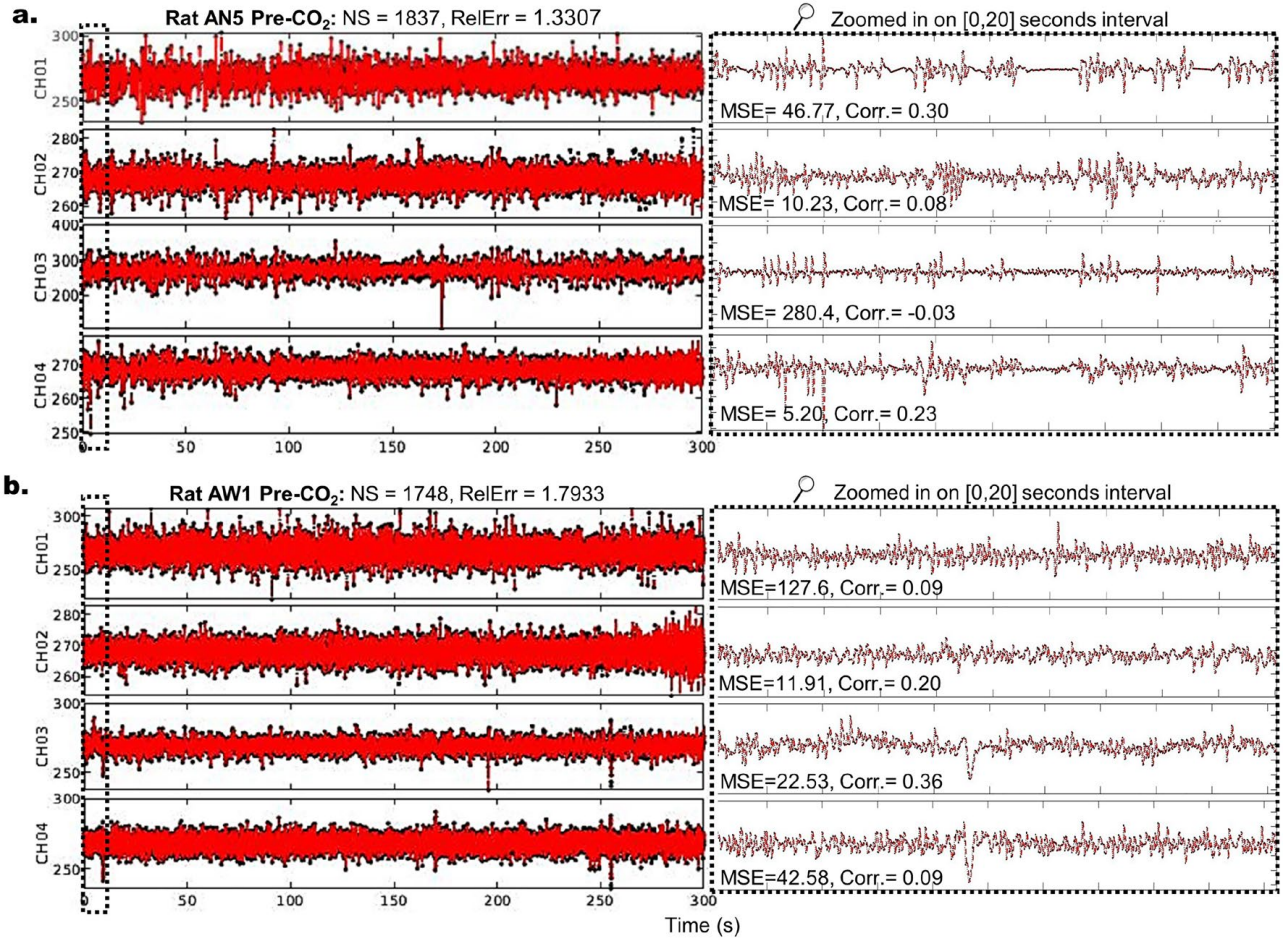
Comparison of ERA Reconstructions for Anesthetized (AN) and Awake (AW) Rats. Panels (**a**) and (**b**) display the ERA reconstructions of recorded time series for AN Rat 5 and AW Rat 1 during the Pre-CO_2_ phase, respectively. Each row corresponds to the time series of a specific channel. In all plots, raw data is shown in black, while ERA reconstructions are shown in red. The left panels present the complete phase reconstruction, with the number of stacks (NS) and relative error (RelErr) annotated above. The right panels provide a closer look at a selected segment, detailing the mean square error (MSE) and Pearson correlation coefficient (Corr) for each channel.

### Compliance with ethical standards

All methods detailed in this research were approved by the Institutional Animal Care and Use Committee (IACUC) of the University of Texas at Arlington and in strict accordance with the ARRIVE (Animal Research: Reporting of In Vivo Experiments) guidelines (https://arriveguidelines.org).

## Results

We have developed a novel data-driven pipeline for the reconstruction of brain LFP signals recorded from rats, capturing brain activity in four distinct regions both before and after anesthesia. Our initial experiments involved anesthetized rats (AN) and awake rats (AW), each recorded for 5 min. Additionally, we investigated the recorded signals for both groups during exposure to CO_2_ (euthanasia). In the following sections, we will delve into the technical and statistical characteristics of our reconstructions across the various phases of the recording.

### Accurate ERA reconstructions of LFP signals

**Table 1:**
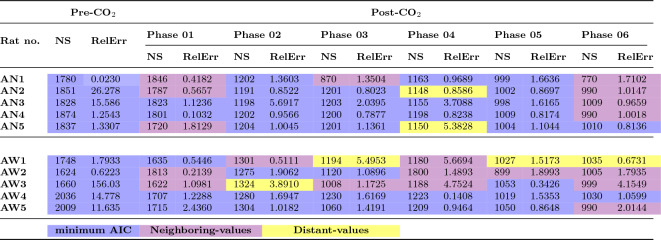
Number of stacks (NS), relative error (RelErr), and NS selection criterium.

Figure 4 showcases visualizations of the ERA reconstructions for two rats, AN5 and AW1, to demonstrate the quality of these reconstructions. In all plots, raw data is depicted in black, and the ERA reconstruction is shown in red. The left panels depict the reconstruction of the time series for the entire 5-min phase, while the right panels zoom in on a much shorter 20-s interval. The corresponding number of stacks (NS) and relative error (RelErr) are annotated at the top. Each row corresponds to a single channel, with the mean square error (MSE) and Pearson correlation coefficient (Corr) annotated for each channel. Overall, ERA achieves excellent reconstructions for all time series, but the required NS varies among rats and phases, potentially reflecting behavioral differences.

### Variation of Number of Stacks (NS) across phases

Table 1 details the selected NS across various experimental phases and rat groups, along with the corresponding relative error and the criteria for NS selection, indicated by a color code. Before the administration of CO_2_, both rat groups show similar NS values. The administration of CO_2_ starts in Phase 1; however, due to the delayed effect of CO_2_, a significant reduction in NS values is noted only in Phase 2. Subsequently, NS values continue to decrease, reaching their lowest in Phase 6.

After CO_2_ administration, NS values begin to show more variability, especially within the AW group, even though NS decreases for both groups. Notably, the AN group’s NS values are generally lower, yet they tend to align more closely with the AW group’s values post-CO_2_. This variation in NS suggests differences in performance across different experimental conditions. Various statistical analysis techniques, such as Mann-Whitney and Welch t-tests, support this claim, as discussed later in Section Results ’*Biological validation of NS decay and last fight during euthanasia*’. For a comprehensive statistical analysis, including relative error calculations and comparisons of Change Points (CPs) using BEAST, see SI-(3-5).

### Determining NS for segment reconstruction via ERA

The NS is a crucial parameter for ERA and is instrumental in the construction of Hankel Matrices and significantly impacting the Relative Error (RelErr). Increasing NS typically leads to a reduction in RelErr, indicating a stronger model fit and an enhanced ability to identify complex patterns within the data. Theoretically, a sufficiently large NS allows for near-perfect reconstruction of the time series. However, this could result in overfitting, which is undesirable. To identify the optimal model, we utilize the Akaike Information Criterion (AIC), a statistical measure that evaluates the trade-off between model complexity and accuracy by considering the goodness of fit and the number of parameters. A lower AIC value denotes a more favorable balance, making it the preferred model.

**Figure 5:**
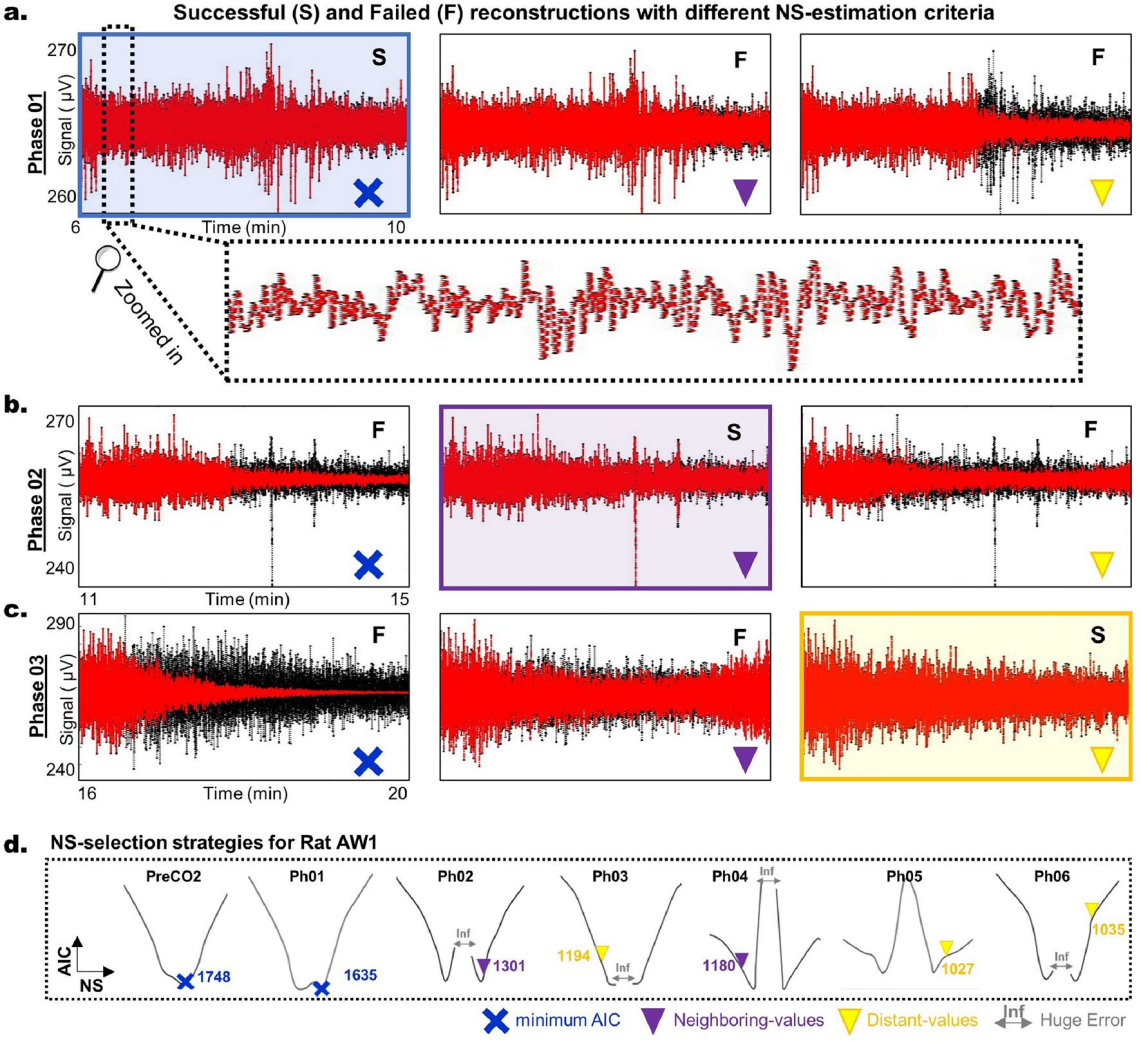
AIC-NS selection. This figure displays the 3-case AIC selection strategies for NS. All data is taken from AW1. Panel (**a**) illustrates the basic case where the NS is the minimum AIC in phase 01 alongside a zoomed-in view of the reconstruction. Panel (**b**) presents the Neighboring-values scenario where the NS is not the minimum AIC, but its proximity to the minimum AIC is notable in phase 02. Panel (**c**) explains the last case, ‘Distant values’, where AIC patterns differ from previous cases, in phase 03. Panel **d** displays the relationship between AIC and the NS for all phases: Pre-$CO_2$ and Ph01 phases indicate the NS =min(AIC) (in blue). Ph02 and Ph04 phases indicate the NS = Neighboring-values (in purple). Ph03, Ph05, and Ph06 phases indicate the NS = Distant-values (in yellow).

Our approach to selecting the appropriate NS starts with a coarse grid search, evaluating various NS values in the form $NS = 2^k$, where $k = 1, \ldots , 13$, and assessing their corresponding AIC values. We then proceed with a refined search in areas that exhibit low AIC values. Finally, we choose the NS through three nested conditional checks: Initially, we visually inspect the reconstruction achieved by NS* (the NS yielding the lowest AIC or min-AIC). If satisfactory, we select $NS = NS^*$. Figure 5a illustrates a successful application of this strategy. If unsatisfactory, we consider neighboring values ($NS = NS^* \pm 10$). Figure 5b depicts a case where the min-AIC strategy fails, but the neighboring-values strategy succeeds. If this method also proves insufficient, we explore NS values further from NS* and its adjacent values until we attain (i) visually satisfactory reconstructions and (ii) RelError comparable to those of similar rat groups and phases (distant-values strategy). Figure 5c presents a scenario where this last-resort strategy is adopted.

Figure 5d illustrates the NS-selection strategies for each phase of Rat AW1: min-AIC was employed in the Pre-CO_2_ phase and Phase 1, indicated by a blue cross, while neighboring-values were chosen for Phases 2 and 4, marked by purple triangles. Distant-values were selected for the other phases, shown with yellow triangles. Finally, Table 1 adopts this color-coding to summarize the selection strategy for each rat in each phase, with the min-AIC strategy, highlighted in blue, being the most frequently utilized.

### Robustness of segment reconstructions with ERA

**Figure 6:**
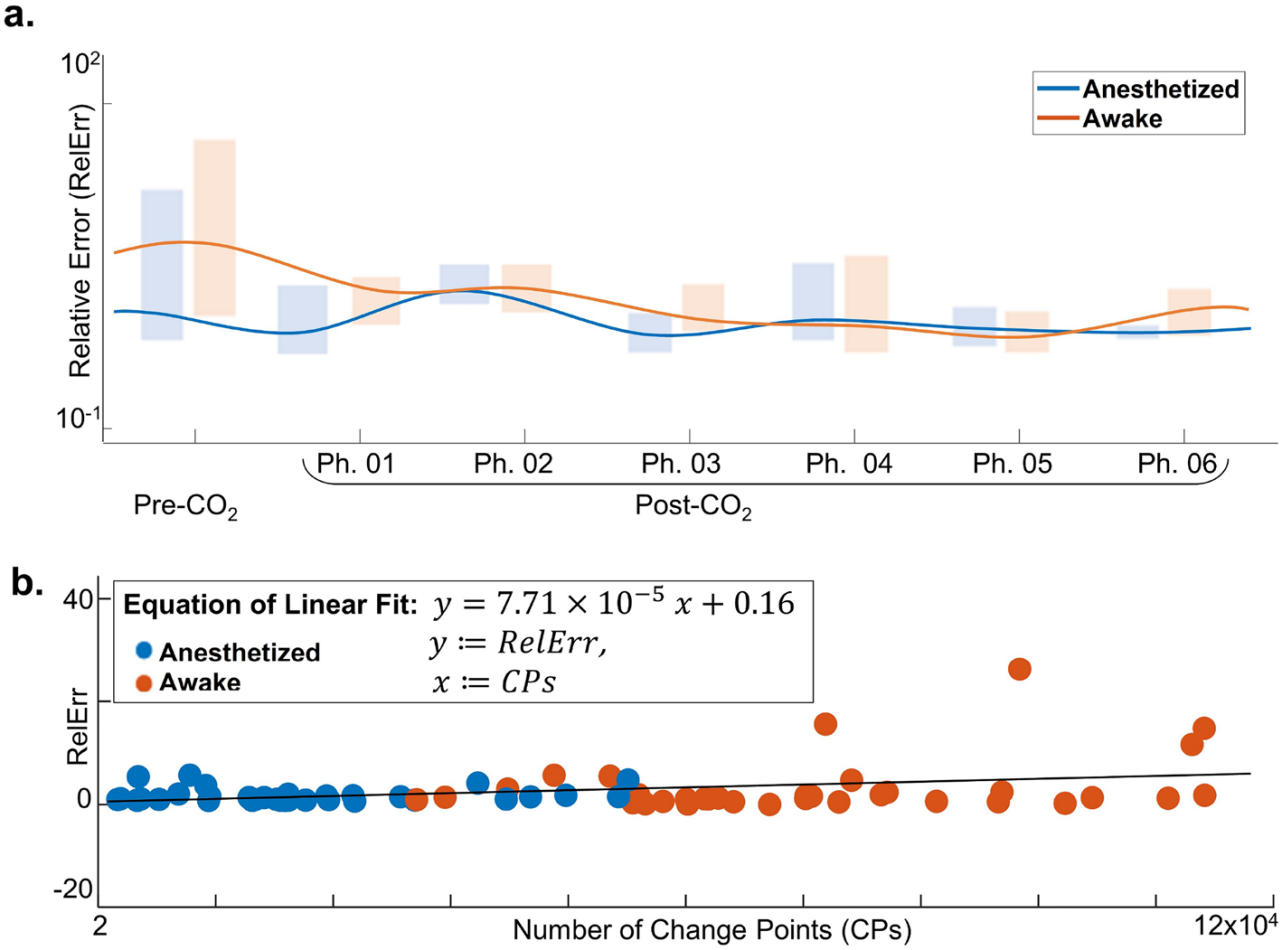
Robustness of segment reconstructions with ERA. Panel (**a**) illustrates the Relative Error (RelErr) of our reconstructions for anesthetized rats (AN, in blue) alongside awake rats (AW, in red). Shaded rectangles indicate the boxplot of RelErr for rats within each group for that phase. Panel (**b**) explores the relationship between the RelErr of a segment and its number of Change Points (CPs). One outlier point (67763, 156.04) was not depicted, and the linear fit equation is given by $y = 7.71\times 10^{-5}x + 0.16$. This demonstrates that RelErr remains consistent across a broad x-axis range from 2 to $1.2\times 10^{5}$, indicating that the number of CPs does not markedly affect the quality of reconstruction.

This subsection aims to demonstrate the robustness of segment reconstructions using ERA in the face of data heterogeneity. Firstly, we establish that the Relative Error (RelErr) remains consistent across all groups and phases. Secondly, we show that the RelErr is not affected by the number of CPs identified in different segments through BEAST. Figure 6a shows that RelErr for both AW and AN groups is comparable across most phases, with the group averages per phase (blue and orange lines) nearly overlapping, except in the pre-CO_2_ phase. Shaded rectangles represent boxplots for each group. All RelErr values are provided in Table 1. Notably, RelErr for AW3 in Phase 1 is an outlier; excluding it suggests no significant statistical differences between the groups. Therefore, variations in NS cannot simply be attributed to changes in the quality of segment reconstructions.

The number of CPs identified per segment by BEAST varied significantly. This variability is seen across rats, channels, and time intervals, reflecting the unique brain activities and responses of individual subjects. The variation arises from the specific brain regions each channel targets and the fluctuating nature of brain activity. Nonetheless, ERA successfully produced consistent reconstructions across all segments. Figure 6b plots the RelErr as a function of the number of CPs. Despite a broad range on the x-axis, from 2 to $1.2 \times 10^5$, RelErr remained relatively unchanged, suggesting that the quantity of CPs does not markedly influence the quality of reconstruction.

### Biological validation of NS decay and last fight during euthanasia

ERA identifies governing equations for the underlying, non-observable high-dimensional system by determining matrices *A* and *B*. It also reveals the system’s impact on observations through matrix *C* and enables the reconstruction of the system’s dynamics. For more information, see Eq. (1) and the Methods section. Although these concepts are hard to interpret, we provide a validation for NS, a crucial parameter that must be adjusted for reconstructing more intricate time series.

**Figure 7:**
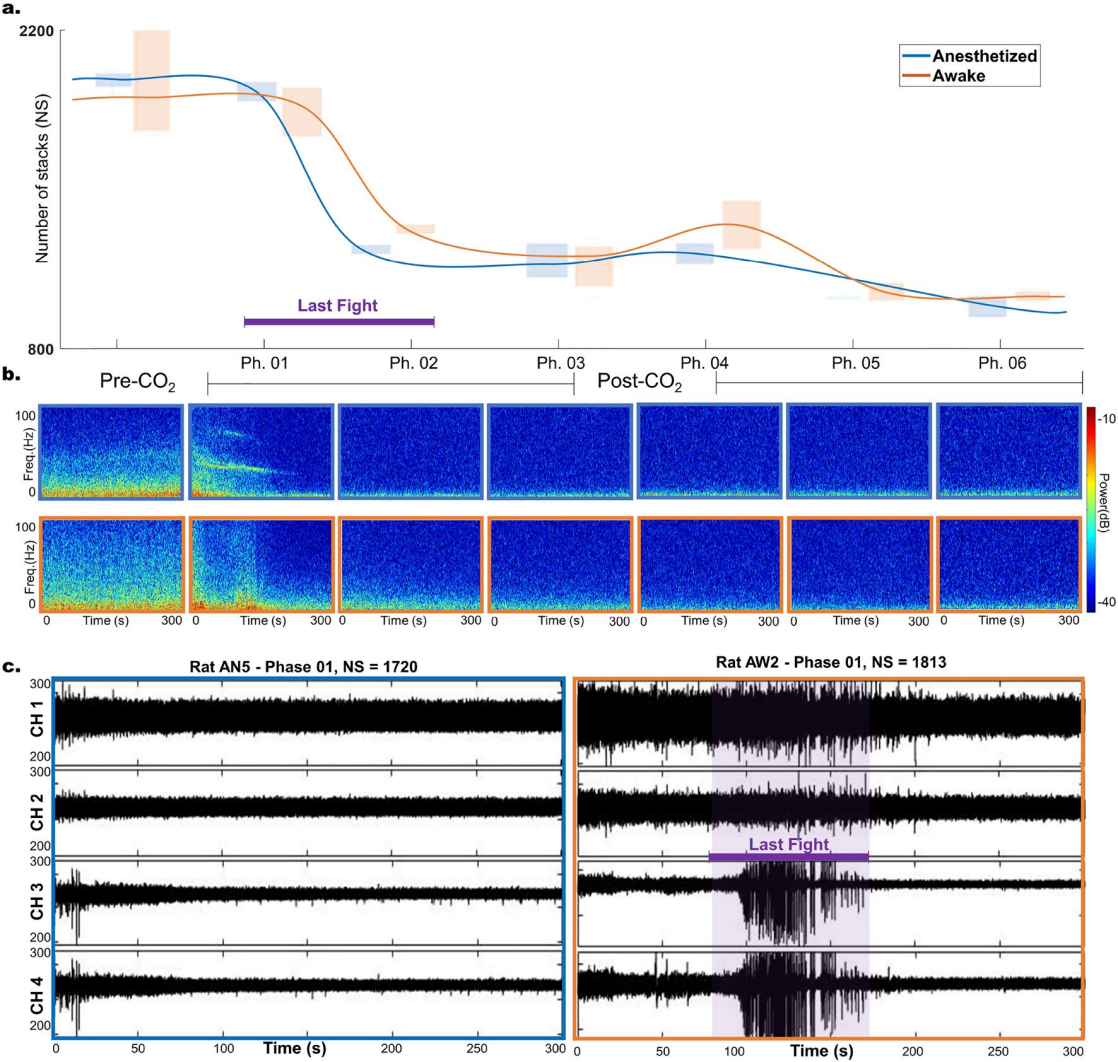
Number of stacks (NS) analysis. Panel (**a**) illustrates the variation in NS for the anesthetized (AN) in comparison to the Awake (AW) rats. Shaded rectangles represent the boxplot of NS values for rats within the same group during that phase. Panel (**b**) demonstrates the windowed power spectra for the corresponding phases, calculated using a 30-s sliding window. The power spectra were averaged per channel and per rat group, with the AN group at the top and the AW group at the bottom. Power values in dB were represented by a colormap on the right (High: red $\rightarrow$ Low: blue). Panel (**c**) reveals distinct neural activity patterns in rats AN5 and AW2 during phase 01. Panel (**c**) displays reduced brain activity in rat AN5, while rat AW2 exhibits heightened activity between 90 and 170 of phase 01, indicating a unique neurological state elicited by stimulus or behavior.

As Fig. 7a shows, NS decreases with CO_2_ application, reflecting a reduction in brain function and the consequent death of the rats. This finding is consistent with standard windowed power spectrum analysis, illustrated in Fig. 7b. Here, a 30-s sliding window calculates and averages the spectrum across phases and rat groups, with the frequency power (in dB) shown through a color map. The dominance of red at lower frequencies in the Pre-CO_2_ phase for awake rats signifies more active brain function than in the anesthetized group. Subsequently, the power across all frequencies diminishes for both groups, albeit at differing rates, throughout the euthanasia.

Awake rats also typically display a greater resilience to death than their anesthetized counterparts, likely due to unimpaired protective mechanisms. This difference is evidenced by significant disparities in NS values between groups during Phases 1 and 2, confirmed by Mann-Whitney U test results with p-values less than 0.007 for both phases, and Welch t-tests showing p-values of 0.048 for Phase 1 and 0.0002 for Phase 2. Additionally, a Welch t-test examining the absolute differences between Phases 1 and 2 across groups indicates a more pronounced decrease in NS for the AN group, with a p-value of 0.0006.

Lastly, LFP patterns in awake rats suggest the occurrence of a ’last fight’ phenomenon-an unexpected surge in brain signal complexity during the euthanasia process. Figure 7c compares the time-series data for two rats, AN5 and AW2, and highlights the distinct patterns in their brain activity. Specifically, AW2’s brain activity exhibited a notable surge between the highlighted intervals, potentially indicating heightened neural activity in response to a specific stimulus or behavioral response. Conversely, AN5’s brain activity showed a sharp decline, leading to cessation, suggesting a different physiological or neurological state.

## Discussion

In this work, we introduce a novel computational framework designed to accurately reconstruct Local Field Potential (LFP) signals recorded from both anesthetized and awake rats before and during CO_2_ euthanasia. We utilize the Bayesian Estimator of Abrupt Change, Seasonality, and Trend (BEAST) for efficient downsampling of the recorded signals and apply the Eigensystem Realization Algorithm (ERA) to derive linear governing equations capable of reproducing the observed data.

Our approach highlights the benefits of our chosen downsampling method and the precision of ERA reconstructions. The Number of Stacks (NS) is a critical parameter for ERA that, when increased, typically results in decreased Relative Error (RelErr), indicating a stronger model fit and improved detection of complex patterns within the data. We detail how NS can be optimally selected to balance model accuracy and parameter count. Additionally, we underscore the robustness of segment reconstructions using ERA against data heterogeneity. We demonstrate that the Relative Error remains stable across all groups and phases and is unaffected by the variability in the number of Change Points (CPs) identified through BEAST.

Finally, we provide a biological validation of NS, showing its decline with the application of CO_2_, mirroring a decrease in brain function and leading to the eventual demise of the rats. This finding is consistent with standard windowed power spectrum analysis applied to the data. Furthermore, the brain signals of awake rats retain their complexity for a longer period, suggesting a “last fight” response, whereas anesthetized brains exhibit a quicker transition to reduced activity due to anesthesia. Nevertheless, the specific mechanisms and factors contributing to the observed increase in complexity during the “last fight” phase are not fully understood, calling for further research.

### Methodological challenges and limitations

The field of dimensionality reduction offers various methods to reduce the complexity and size of data, such as Proper Orthogonal Decomposition, Balanced Truncation, Balanced Proper Orthogonal Decomposition, the Eigensystem Realization Algorithm (ERA), and the Dynamic Mode Decomposition (DMD). ERA has gained significant attention in the scientific community, and researchers have been exploring new techniques to address the challenges and complexities associated with real-world data^20^. In our study, we overcame several specific issues while applying ERA to our electrophysiological data with the assistance of the BEAST algorithm.

Our dataset included 35 min of LFP recordings from four brain regions in ten rats, resulting in a substantial dataset (approximately 6.5 million data points per channel per rat). When attempting to directly apply ERA to an entire 35-min recording, MATLAB became unresponsive after six days. Even dividing a single channel’s time series into 5 min intervals took up to three days and occasionally led to out-of-memory errors. Handling the growing Hankel matrices, crucial for ERA, presented formidable challenges.

Applying ERA directly to our extensive dataset proved infeasible due to processing time constraints. Even with more powerful machines, the estimated processing time for all the rats’ data exceeded 89 days, which was impractical. To overcome this, we turned to the BEAST algorithm combined with interpolation. This approach involved detecting change points in the data, simulating the original dataset with fewer points, and then further reducing them through interpolation. Detecting points using BEAST took about six hours per rat (four channels) on our server, totaling sixty hours for all rats. By leveraging BEAST and interpolation, we efficiently reduced the data size and successfully applied ERA within a reasonable time frame.

One major limitation of the ERA formulation in our study is its omission of external stimuli, a factor potentially more impactful on awake rats. We plan to refine our model in future research by introducing a term for time-varying external stimuli, $\xi (k)$, independent of the system’s state variables *x* and *y*, into Eq. (1). This addition aims to better account for potential differences in how external influences affect awake versus anesthetized rats, thereby enhancing our understanding of the dynamics observed in our experiments. To accurately incorporate this change, empirical data on external stimuli for identifiability will be necessary, alongside modifications to include $\xi (k)$ in the estimation of matrices *A*, *B*, and *C*. Despite this limitation, our experimental setup, involving the euthanasia of both rat groups in a controlled CO_2_ chamber environment, helps minimize external variability, lending support to the validity of our initial findings.

### Biological insights and future work

In future studies, our aim is to apply our model to publicly available datasets, such as the Allen electrophysiology recordings^21^, to validate further the efficacy and applicability of the ERA reconstruction method across various rodent datasets. The complexity identified by ERA and the requisite number of stacks underscores the need for continued research. Additionally, the mechanisms underlying the “last fight” phase in rodents are not fully understood, necessitating further investigation to elucidate these phenomena.

Two recent studies in rodents shed light on the physiological underpinnings of decapitation and anoxia, offering insights into consciousness and brain function under extreme conditions. The study by Rijn et al. documented a rapid decrease in EEG power following decapitation, suggesting an immediate loss of consciousness and supporting the ’wave of death’ concept that marks the life-to-death transition^22^. Zandt et al.’s research on neural dynamics during anoxia explained the ’wave of death’ resulting from membrane potential oscillations due to the cessation of sodium-potassium pump activity^23^. Applying ERA in these contexts could provide further validation and insights, enhancing our understanding of brain activity under such extreme conditions. In humans, research into heightened brain activity following cardiac arrest suggests the possibility of memory replay after death, as evidenced by observed gamma wave patterns associated with memory and consciousness. This opens up new avenues for exploring near-death experiences and the intricate processes of memory and consciousness as life comes to an end^24^. Leveraging ERA for real-time monitoring of brain activity, especially in ICU settings, could serve as an early warning system for healthcare professionals. This application offers the potential to improve patient care by providing vital insights into patients’ neurological conditions during critical phases. Pursuing this research direction promises to deepen our understanding of near-death experiences, consciousness, and the physiology of human mortality.

### Supplementary Information

Supplementary Information.

## Data Availability

Raw LFP signal data are available in the Headache Journal at https://headachejournal.onlinelibrary.wiley.com/doi/10.1111/head.14506^14^. The dataset utilized in this study is derived from^14^, with all pertinent data included within the article and/or its SI. Furthermore, MATLAB demonstration scripts similar to those employed in our analysis can be found in our GitHub Repository at https://github.com/kza4256/ERA-Demo.
