# Supplementary material for: Reconstructing rodent brain signals during euthanasia with eigensystem realization algorithm (ERA)
Source: Sci Rep. 2024 May 28;14:12261. doi: 10.1038/s41598-024-61706-y (PMC11133335; doi:10.1038/s41598-024-61706-y)
Supplement: Supplementary file 1 — Supplementary Information. [file 41598_2024_61706_MOESM1_ESM.pdf]

Supplemental Information

SI-1. BEAST vs. uniform down-sampling

Fig. 1a compares the simple uniform down-sampling (SUD, in blue) to BEAST (in green) across a 1-second interval, with parameters adjusted so the number of change points (CPs) aligns with points in uniform discretization ($n = 20$). Initially, in Fig. 1b, BEAST exhibits a single, prolonged decreasing trend, in contrast to SUD's three separate trends within the same timeframe—two of which unjustifiably rise. Such frequent, conflicting trends in SUD throughout the series result in a distorted signal representation. SUD's sensitivity to outliers further skews the depiction, introducing erratic changes misrepresenting the system. Additionally, downsampling tends to obscure detailed patterns. In summary, although BEAST may not be essential for every dataset, it significantly enhances our analysis by providing a more reliable and precise depiction of trends, especially by circumventing the misleading trends often produced by SUD.

SI-2. Creating optimal and uniform time discretizations

The system identification technique we employ for reconstructing brain signals relies on evenly spaced time measurements across all channels [1, 2]. However, the time intervals between change points (CPs) obtained from the BEAST algorithm can significantly vary within a channel. Furthermore, CPs in one channel often do not align with CPs in other channels, as illustrated in Fig. 2a. Our goal is to establish a uniform time discretization that remains relatively coarse while ensuring the time points are not significantly distant from the identified CPs.

Our approach to determining the optimal sampling time is as follows: First, we combine the occurrence times of CPs from all channels, sorting and removing any duplicates (top green plot in Fig. 2b). In a uniform discretization, we have $t_{j+1} = t_j + \alpha$, where $\alpha > 0$. Since the set of CPs is fixed, we can define a misalignment function $M(\alpha, \texttt{CPs})$ between the CP times and the t_j times. We empirically seek a balance between (i) large α values (for coarse sampling) and (ii) minimal misalignment with respect to CPs (which typically decreases with α). As the curves associated with (i) and (ii) are increasing and decreasing, respectively, their intersection (typically around $\alpha = 0.01$) yields a coarse, uniformly sampled set of points that closely align with the CPs identified through segmentation analysis (see Fig. 2b, bottom). Following this pre-processing step, the signals are ready for the subsequent time-series reconstruction stage.

SI-3. Statistical analysis of CPs from BEAST segmentation

Tables 1 to 2 provide a comprehensive overview of the necessary number of change points (CPs) for reconstructing brain signals using the BEAST algorithm. The data is organized by measurement channel and covers different experiment phases for both anesthetized (AN) and awake (AW) rats. Generally, the

number of CPs is higher in the Pre-CO_2 phase compared to the euthanasia phases (refer to Table 3). Additionally, the AW group consistently exhibits a greater number of CPs compared to the AN group. In all cases, segmentation analysis conducted with BEAST significantly reduces the required number of time points for representing the time series, while maintaining acceptable R-square values. Table 4 presents the variance analysis (ANOVA) results of the NS for Pre-CO_2 and Post-CO_2 variables. In both cases, the between-group sum of squares (SS) is notably smaller than the within-group SS, indicating that most of the data variability originates from differences within the groups rather than between them. The F-statistics and p-values for both variables are not significant, with p-values well above 0.05, supporting the absence of a significant difference between the groups.

Regarding Table 5, the ANOVA results between the groups for both Pre-CO_2 and Post-CO_2 also reveal non-significant differences in relative error. The between-group SS is considerably smaller than the within-group SS, further confirming the lack of significant distinctions between the groups for these variables.

SI-4. Paired t-test with Pre/Post CO_2 measurements

Table 6 displays the paired t-test results for Pre-CO_2 and Post-CO_2 measurements in both the anesthetized and awake rat groups. The results indicate no significant difference between these measurements within each group. The anesthetized group has a mean difference of -634.60 (SD = 36.30), yielding a t-statistic of -39.09 with a p-value of 0.0000. Similarly, the awake group shows a mean difference of -563.80 (SD = 170.78), resulting in a t-statistic of -7.38 with a p-value of 0.0018. While the AW group exhibits a larger mean difference, the t-test results and p-values demonstrate no significant distinction between the two groups. In summary, there's no significant difference between Pre-CO_2 and Post-CO_2 measurements in both the AN and AW rat groups.

SI-5. Trends and slopes from BEAST

Figures 3-4 display Trend Slopes Analysis for different channels in both the anesthetized (AN) and awake (AW) rat groups. Median slopes, represented by black lines, show the central trend, with gray-shaded areas indicating variability (median $\pm$ standard deviation). Blue and red-filled regions enclose upper and lower bounds containing outliers. Paired t-tests (Tables 7 and 8) assess differences in four channels (CH01-CH04) between Pre-CO_2 and Post-CO_2 measurements for AN and AW groups.

For the AN group, mean differences between Pre-CO_2 and Post-CO_2 measurements are generally small (-0.004 to 0.022), with standard deviations ranging from 0.18 to 0.54. A significant effect is observed in AN1-CH01. Conversely, the AW group's mean differences are small (-0.005 to 0.007) but with larger standard deviations (0.000 to 2.769), indicating more data variability.

In summary, paired t-test results reveal generally insignificant differences between Pre-CO_2 and Post-CO_2 measurements, except for a few cases. Variability across rats and channels suggests that more data is needed to reach definitive conclusions. Mean differences and standard deviations tend to be smaller in the awake group, although the results are less clear due to larger standard deviations and less significant p-values.

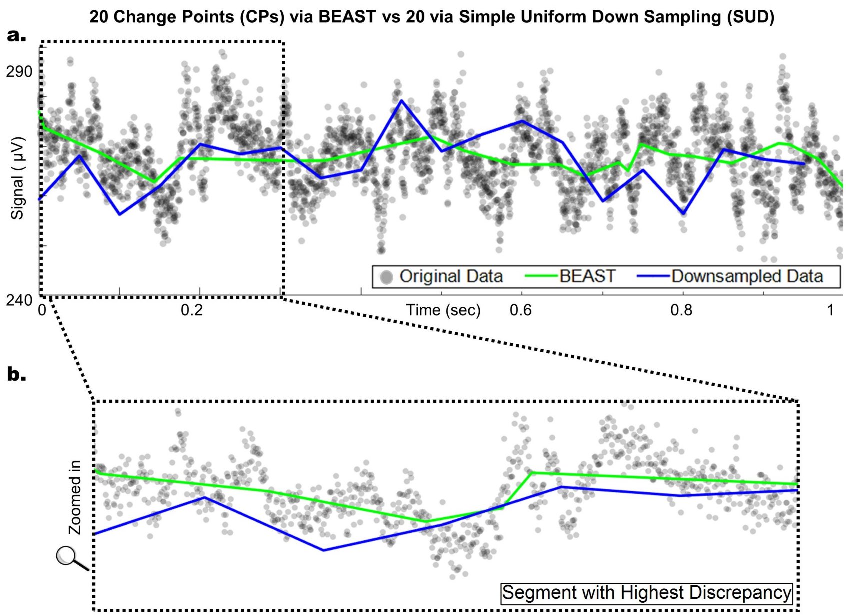

Fig. 1 BEAST vs. Simple Uniform Downsampling (SUD). Panel **a** represents the downsampling of a 1-second interval, with a data sampling frequency of 2999, rat AW1-pre-CO_2 phase. The SUD reconstruction (in Blue) and the `BEAST` (in green) along with the original data (gray-filled dots). Panel **b** illustrates the reconstruction on the zoomed-in interval from 0 to 0.3.

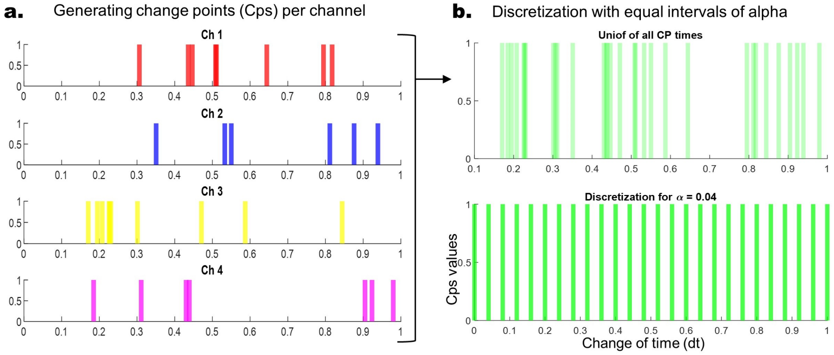

Fig. 2 Schematics for unifying change points across channels after `BEAST` processing. Panel **a** illustrates the change points (`CPs`) generated by `BEAST` for each channel. Due to their non-uniform locations, `CPs` often misalign across channels. Panel **b** demonstrates, at the top, the aggregation of all `CPs` from Panel **a**. Our approach to achieving a coarse uniform sampling involves setting $t_{j+1} = t_j + \alpha$, where $\alpha > 0$. Given the fixed set of `CPs`, we define a misalignment function $M(\alpha, \texttt{CPs})$ that measures the discrepancy between the `CP` locations and the t_j times. We aim to find an optimal balance between choosing large α values for coarse sampling and minimizing misalignment with the `CPs`, which generally diminishes as α increases. The intersecting points of the curves representing these two objectives (usually near $\alpha = 0.01$) identify a set of coarsely, uniformly sampled points that closely match the `CPs` determined through segmentation analysis.

Table 1 BEAST Results for the Anesthetized Group

Rat no.	Channel	$PreCO_2$			$PostCO_2$ - **Euthanasia**																		
		Pre-Euthanasia (0 − 5 min)			**Phase 01** (5 − 10 min)			**Phase 02** (10 − 15 min)			**Phase 03** (15 − 20 min)			**Phase 04** (20 − 25 min)			**Phase 05** (25 − 30 min)			**Phase 06** (30 − 35 min)		
		**no. CPs**	$\mathbf{R^2}$	α	**no. CPs**	$\mathbf{R^2}$	α	**no. CPs**	$\mathbf{R^2}$	α	**no. CPs**	$\mathbf{R^2}$	α	**no. CPs**	$\mathbf{R^2}$	α	**no. CPs**	$\mathbf{R^2}$	α	**no. CPs**	$\mathbf{R^2}$	α
**AN1**	**CH01**	22756	0.9998	0.0099	14117	0.4283	0.0099	2854	0.0245	0.0100	2913	0.0535	0.0099	2904	0.0351	0.0099	2964	0.0535	0.0098	2977	0.0335	0.0099
	**CH02**	25797	0.9964		15076	0.2101		6340	0.4516		6581	0.5163		6494	0.4459		8403	0.5672		8872	0.3606	
	**CH03**	15874	0.8069		12359	0.1115		10626	0.0565		11306	0.1419		10985	0.0124		13643	0.2244		14479	0.2289	
	**CH04**	16940	0.6177		16579	0.1795		15250	0.0455		15541	0.8711		15161	0.2258		17207	0.2546		18253	0.3105	
**Total (pre-alignment)**		81367			58131			35070			36341			35544			42217			44581		
**Total (post-alignment)**		77135			54843			32839			34129			33318			39435			41682		
**AN2**	**CH01**	22373	0.9996	0.0099	12236	0.0129	0.0099	2971	0.0105	0.0099	2969	0.0819	0.0099	2972	0.1398	0.0099	2963	0.1143	0.0098	2381	0.0802	0.0099
	**CH02**	25403	0.8924		19555	0.4235		9617	0.2982		10747	0.3679		11304	0.0782		11526	0.3337		10808	0.3918	
	**CH03**	30433	0.9950		25551	0.4846		12796	0.0826		11265	0.1543		9680	0.1508		9033	0.0792		8552	0.1969	
	**CH04**	26467	0.5837		21611	0.0250		14891	0.0802		13452	0.1622		14061	0.0264		15119	0.0140		15119	0.0140	
**Total (pre-alignment)**		104676			78953			40275			38433			38017			38641			36860		
**Total (post-alignment)**		98411			74064			37655			35926			35681			36205			35180		
**AN3**	**CH01**	13108	0.5525	0.0099	10392	0.0933	0.0098	2986	0.1041	0.0099	10392	0.0587	0.0099	2983	0.0741	0.0099	2995	0.0639	0.0099	2333	0.0637	0.0099
	**CH02**	24523	0.7689		21841	0.4592		12094	0.1720		12145	0.3235		12871	0.1205		13131	0.4451		9894	0.3940	
	**CH03**	25720	1.0006		25261	0.5269		8283	0.0632		7009	0.0859		7013	0.0002		7804	0.0002		5535	0.2022	
	**CH04**	23394	0.8611		18697	0.0937		6732	0.0285		6933	0.2694		8592	0.0055		7843	0.0536		5583	0.1818	
**Total (pre-alignment)**		86745			76191			30095			36479			31459			31773			23345		
**Total (post-alignment)**		81890			71622			27802			26837			29151			29499			21662		
**AN4**	**CH01**	8778	0.3845	0.0095	5914	0.1693	0.0087	3025	0.0590	0.0099	2969	0.0525	0.0076	2986	0.0400	0.0083	2986	0.0607	0.0099	2996	0.0658	0.0075
	**CH02**	21502	0.3077		21815	0.0886		12358	0.1297		9969	0.7775		12768	0.2820		13445	0.4351		16066	0.1813	
	**CH03**	26054	0.8393		25148	0.1399		12008	0.1163		7526	0.2306		9992	0.1302		8193	0.0833		10640	0.1750	
	**CH04**	20687	0.4074		21440	0.3780		15100	0.0434		11189	0.0545		12442	0.2719		10936	0.2055		12544	0.1300	
**Total (pre-alignment)**		77021			74317			42491			31653			38188			35560			42246		
**Total (post-alignment)**		72765			70168			39621			29399			35517			33120			39609		
**AN5**	**CH01**	24934	0.8200	0.0098	11786	0.0071	0.0097	3033	0.0578	0.0099	2988	0.1182	0.0099	2988	0.1182	0.0099	3005	0.0728	0.0098	2397	0.1105	0.0099
	**CH02**	28849	0.6256		19308	0.1520		7952	0.2615		7725	0.0379		7848	0.2462		8324	0.4687		8820	0.3122	
	**CH03**	30600	0.9997		22855	0.1009		7609	0.3646		6664	0.1545		7264	0.0446		7104	0.2071		6724	0.0057	
	**CH04**	26906	0.5662		18214	0.1990		8469	0.2089		6273	0.1800		7108	0.3107		7033	0.1589		7184	0.0505	
**Total (pre-alignment)**		111289			72163			27063			23650			25208			25466			25125		
**Total (post-alignment)**		104584			68062			25182			21899			23412			23631			23319		

Table 2 BEAST Results for the Awake Group

Rat no.	Channel	$PreCO_2$ Pre-Euthanasia $(0-5$ min)			$PostCO_2$ - Euthanasia Phase 01 $(5-10$ min)			Phase 02 $(10-15$ min)			Phase 03 $(15-20$ min)			Phase 04 $(20-25$ min)			Phase 05 $(25-30$ min)			Phase 06 $(30-35$ min)		
		**no. CPs**	R^2	α	**no. CPs**	R^2	α	**no. CPs**	R^2	α	**no. CPs**	R^2	α	**no. CPs**	R^2	α	**no. CPs**	R^2	α	**no. CPs**	R^2	α
**AW1**	**CH01**	30600	0.8929	0.0099	29954	0.4039	0.0099	21150	0.1488	0.0099	16044	0.1106	0.0099	16044	0.1044	0.0098	4283	0.0875	0.0099	2599	0.0076	0.0099
	**CH02**	30460	0.6260		23857	0.5264		19373	0.3732		6926	0.1044		6693	0.0787		8324	0.3881		7249	0.0921	
	**CH03**	30376	0.8832		24354	0.5124		23136	0.2932		20793	0.0866		16874	0.0225		13443	0.0025		11941	0.1117	
	**CH04**	30600	0.9621		24299	0.7977		24294	0.4116		23189	0.1993		22228	0.3125		22076	0.7051		22213	0.2328	
**Total (pre-alignment)**		122036			102464			87953			66952			61839			48126			44002		
**Total (post-alignment)**		114152			96592			83028			63557			58801			45729			41828		
**AW2**	**CH01**	24380	0.9138	0.0099	30449	0.5609	0.0099	26331	0.2056	0.0098	19439	0.0302	0.0099	13285	0.0307	0.0100	11143	0.1264	0.0099	7759	0.0927	0.0099
	**CH02**	24150	0.9191		27942	0.6252		28705	0.5274		22398	0.3259		20099	0.3265		25277	0.2078		23616	0.1077	
	**CH03**	24479	0.9969		22119	0.0445		6591	0.0000		8039	0.0713		7689	0.2174		10303	0.2672		8701	0.2232	
	**CH04**	24480	0.9740		28287	0.6667		29934	0.3643		25972	0.1122		18847	0.2287		23036	0.2242		23129	0.2338	
**Total (pre-alignment)**		97489			108797			91561			75848			59920			69759			63205		
**Total (post-alignment)**		91333			102272			86622			71916			56795			65985			59799		
**AW3**	**CH01**	2403	0.1045	0.0099	3046	0.1181	0.0099	2967	0.0488	0.0098	2967	0.0672	0.0099	2971	0.0695	0.0099	2974	0.0352	0.0099	2263	0.0002	0.0100
	**CH02**	22608	0.7600		29616	0.7022		30268	0.5442		30375	0.7190		30373	0.6442		30432	0.6047		23099	0.7400	
	**CH03**	23358	0.9198		30099	0.4803		29101	0.3202		26107	0.3018		16874	0.1574		22967	0.1614		16703	0.0587	
	**CH04**	23248	0.8817		29240	0.5196		26473	0.1692		24982	0.2060		18207	0.2084		12515	0.0017		12515	0.1528	
**Total (pre-alignment)**		71617			92001			88809			84431			68425			68888			54580		
**Total (post-alignment)**		67763			87126			84107			80205			65089			65502			52304		
**AW4**	**CH01**	30251	0.7477	0.0099	29447	0.2196	0.0099	10200	0.0641	0.0097	10200	0.0641	0.0099	3037	0.1383	0.0098	3066	0.0165	0.0097	2772	0.1066	0.0099
	**CH02**	30385	0.7537		28002	0.3035		17636	0.2008		24517	0.4109		24631	0.3106		23692	0.3041		20172	0.2878	
	**CH03**	30590	0.9951		30421	0.4546		26471	0.2011		20155	0.0539		12559	0.2024		12389	0.1318		11832	0.1049	
	**CH04**	30414	0.6173		30472	0.8334		30376	0.5762		30180	0.5155		29711	0.3797		28440	0.2681		22779	0.0701	
**Total (pre-alignment)**		121640			118342			84683			85052			69938			67587			57555		
**Total (post-alignment)**		114111			111039			80228			80686			66556			64281			54724		
**AW5**	**CH01**	30423	0.3086	0.0098	27218	0.0507	0.0099	14235	0.0197	0.0098	5305	0.0226	0.0097	3968	0.0166	0.0098	3258	0.0535	0.0099	2648	0.0670	0.0100
	**CH02**	30104	0.2256		16515	0.2642		10618	0.2744		11188	0.2634		11147	1.3906		11220	0.0637		9618	0.2967	
	**CH03**	29973	0.2291		29453	0.0285		22402	0.2018		15209	0.1344		15016	0.1006		17262	0.0563		11856	0.0563	
	**CH04**	30161	0.5505		29670	0.5197		26519	0.2308		20623	0.2139		19579	0.1401		17854	0.1367		14121	0.1747	
**Total (pre-alignment)**		120661			102856			73774			52325			49710			49594			38243		
**Total (post-alignment)**		113079			96900			70041			49523			47097			46958			36163		

Table 3 Comparison between the number of change points generated via `BEAST` for the Anesthetized and Awake groups for Pre/Post-CO_2

Group	Number of Change Points Post Alignment					
	Rat No.	Pre-CO_2	Post-CO_2	Total	Percentage% per minute	
					Pre-CO_2	Post-CO_2
Anesthetized	**AN1**	77135	236246	313381	15.42	78.74
	**AN2**	98411	254711	353122	19.68	84.90
	**AN3**	81890	206573	288463	16.37	68.85
	**AN4**	72765	247434	320199	14.55	82.47
	**AN5**	104584	185505	290089	20.91	61.83
Awake	**AW1**	114152	389535	503687	22.83	12.98
	**AW2**	91333	443389	534722	18.26	14.77
	**AW3**	67763	434333	502096	13.55	14.47
	**AW4**	114111	457514	571625	22.82	15.25
	**AW5**	113079	346682	459761	22.61	11.55

Table 4 One-way ANOVA stat for the number of stacks (NS) for both groups *Pre/Post* CO_2.

$Pre - CO_2$	Sum of Squares(SS)	Deg. of Freedom(DF)	Mean Square(MS)	F-statistic(F)	p-value
**Between Groups**	1210.9	1	1210.9	0.0252	0.8746
**Within Groups**	1.5805e+06	33	47894	NaN	NaN
**Total**	1.5817e+06	34	NaN	NaN	NaN

$Post - CO_2$	Sum of Squares(SS)	Deg. of Freedom(DF)	Mean Square(MS)	F-statistic(F)	p-value
**Between Groups**	4295.7	1	4295.7	0.0005	0.98193
**Within Groups**	2.7204e+08	33	8.2435e+06	NaN	NaN
**Total**	2.7204e+08	34	NaN	NaN	NaN

Table 5 One-way ANOVA stat for the Relative Error (RelErr) for both groups *Pre/Post* CO_2.

$Pre - CO_2$	Sum of Squares(SS)	Deg. of Freedom(DF)	Mean Square(MS)	F-statistic(F)	p-value
**Between Groups**	2759.6	1	2759.6	0.4073	0.5277
**Within Groups**	2.2354e+05	33	6773.9	NaN	NaN
**Total**	2.263e+05	34	NaN	NaN	NaN

$Post - CO_2$	Sum of Squares(SS)	Deg. of Freedom(DF)	Mean Square(MS)	F-statistic(F)	p-value
**Between Groups**	0.4590	1	0.4590	0.0022	0.9631
**Within Groups**	6964.2	33	211.04	NaN	NaN
**Total**	6964.7	34	NaN	NaN	NaN

Table 6 The statistical analysis of the number of stacks (NS) & the relative error (RelErr)

Group	Statistical Analysis of NS: Pre/ Post CO_2			
	**Mean difference**	**Std deviation**	**t-statistic**	**p-value**
**AN**	-634.60	36.30	-39.09	2.56e-06
**AW**	-518.60	198.96	-5.83	0.0043
	**Statistical Analysis of RelErr: Pre/ Post CO_2**			
	**Mean difference**	**Std deviation**	**t-statistic**	**p-value**
**AN**	-6.92	11.26	-1.37	0.2411
**AW**	-35.17	65.67	-1.20	0.2972

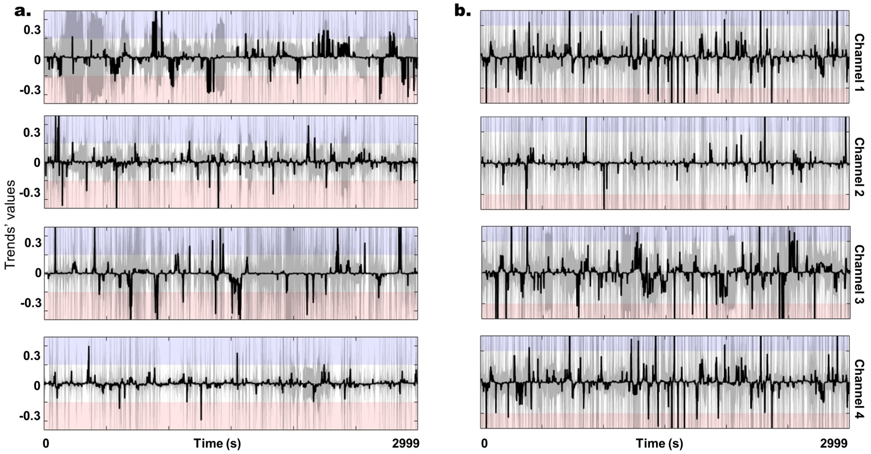

Fig. 3 Trends' slopes generated via `BEAST` **for** $Pre - CO_2$**.** Trend Slopes visualization for both rats' groups $Pre-CO_2$, each row represents one channel. Panel **a** anesthetized (AN), and panel **b** awake (AW) rats. The median slopes of the trends(solid black line), the range of uncertainty/ variability around median ± standard deviation (gray-shaded areas), and the upper and lower bounds containing the outliers (blue and red-filled regions, respectively).

References

[1] Brunton, B.W., Johnson, L.A., Ojemann, J.G., Kutz, N.: Extracting spatial–temporal coherent patterns in large-scale neural recordings using dynamic mode decomposition. Journal of Neuroscience Methods **258**, 1–15 (2016). https://doi.org/10.1016/j.jneumeth.2015.10.010

[2] White, A., Tolman, M., Thames, H.D., Withers, H.R., Mason, K.A., Transtrum, M.K.: The limitations of model-based experimental design

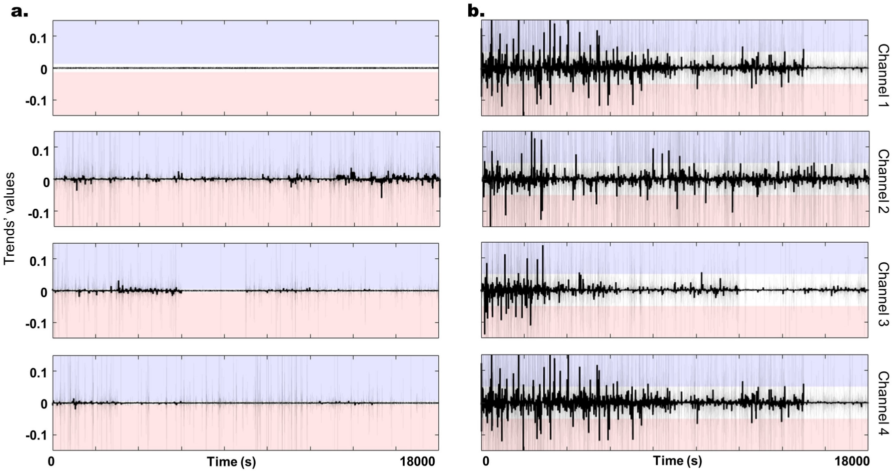

Fig. 4 Trends' slopes generated via BEAST for $Post - CO_2$. Trend Slopes visualization for both rats' groups $Post - CO_2$, each row represents one channel. Panel **a** anesthetized (AN), and panel **b** awake (AW) rats. The median slopes of the trends(solid black line), the range of uncertainty/ variability around median ± standard deviation (gray-shaded areas), and the upper and lower bounds containing the outliers (blue and red-filled regions, respectively).

and parameter estimation in sloppy systems. PLoS computational biology **12**(12), 1005227 (2016)

Table 7 The paired t-test for CPs of the Anesthetized group.

RAT no.	Channel	paired t-test (Pre /post- CO2)			
		**Mean diff.**	**Std deviation**	**t-statistic**	**p-value**
**AN1**	CH01	0.0215	0.1786	6.5907	1e-10
	CH02	0.0003	0.2341	0.0903	0.9280
	CH03	-0.0015	0.3466	-0.2500	0.8025
	CH04	-0.0007	0.3466	-0.1192	0.9050
**AN2**	CH01	0.0017	0.1883	0.5047	0.6137
	CH02	0.0005	0.4569	0.0717	0.9427
	CH03	-0.0023	0.4258	-0.3004	0.7638
	CH04	0.0010	0.2634	0.2105	0.8332
**AN3**	CH01	0.0008	0.5426	0.0843	0.9327
	CH02	0.0006	0.2586	0.1447	0.8849
	CH03	-0.0006	0.1817	-0.1854	0.8529
	CH04	-0.0009	0.3002	-0.1683	0.8662
**AN4**	CH01	-1e-5	0.0044	-0.2010	0.8406
	CH02	0.0003	0.0831	0.2019	0.8399
	CH03	0.0004	0.4800	0.0558	0.9554
	CH04	-0.0006	0.0943	-0.3581	0.7202
**AN5**	CH01	-0.0043	0.3876	-0.6194	0.5356
	CH02	-0.0015	0.2527	-0.3354	0.7373
	CH03	-0.0033	0.3284	-0.5641	0.5726
	CH04	-0.0004	0.1650	-0.1393	0.8891

Table 8 The paired t-test for for CPs of the Awake group.

RAT no.	Channel	paired t-test (Pre /post- CO2)			
		**Mean diff.**	**Std deviation**	**t-statistic**	**p-value**
**AW1**	CH01	0.0047	1.6071	0.1614	0.8717
	CH02	1e-5	0.3756	0.0060	0.9951
	CH03	-0.0030	0.5123	-0.3227	0.7469
	CH04	0.0003	0.6328	0.0314	0.9749
**AW2**	CH01	-0.0020	1.6019	-0.0715	0.9429
	CH02	0.0006	0.5266	0.0640	0.9489
	CH03	0.0067	0.4652	0.8000	0.4237
	CH04	0.0044	0.5177	0.4660	0.6412
**AW3**	CH01	1e-6	1e-6	1.4861	0.1373
	CH02	-0.0054	2.7692	-0.1085	0.9135
	CH03	-0.0013	0.6080	-0.1186	0.9055
	CH04	0.0007	0.5827	0.0747	0.9404
**AW4**	CH01	0.0018	0.7482	0.1390	0.8894
	CH02	-0.0008	0.5324	-0.0893	0.9288
	CH03	-0.0056	0.5579	-0.5496	0.5825
	CH04	0.0019	0.4720	0.2252	0.8217
**AW5**	CH01	0.0015	0.5414	0.1593	0.8734
	CH02	-0.0018	0.2512	-0.4060	0.6847
	CH03	-0.0028	0.3819	-0.4028	0.6870
	CH04	-0.0024	0.5664	-0.2336	0.8152
